# Cytotoxic Activity of Curcumin towards CCRF-CEM Leukemia Cells and Its Effect on DNA Damage

**DOI:** 10.3390/molecules14125328

**Published:** 2009-12-17

**Authors:** Yu Kong, Wei Ma, Xia Liu, Yuangang Zu, Yujie Fu, Nan Wu, Lu Liang, Liping Yao, Thomas Efferth

**Affiliations:** 1 Key Laboratory of Forest Plant Ecology, Ministry of Education, Northeast Forestry University, Harbin 150040, China; E-Mails: kongyu820202@yahoo.com.cn (Y.K.); zygorl@vip.hl.cn (Y.Z.); lx20030135@yahoo.com.cn (X.L.); bao_doubao@yahoo.com.cn (N.W.); luliang20090320@yahoo.com.cn (L.L.); ylp2003@126.com (L.Y.); 2 Engineering Research Center of Forest Bio-preparation, Ministry of Education, Northeast Forestry University, Harbin 150040, China; 3 Heilongjiang University of Traditional Chinese Medicine, Harbin 150040, China; E-Mail: maboshi99@yahoo.com.cn (W.M.); 4 Department of Pharmaceutical Biology, Institute of Pharmacy, University of Mainz, 55099 Mainz, Germany; E-Mail: t.efferth@dkfz.de (T.E.)

**Keywords:** curcumin, activity, CEM, DNA damage

## Abstract

The cytotoxic activity of curcumin towards CCRF-CEM human T-cell leukemia cells was measured by the MTT assay. Tumor cells were more sensitive to the cytotoxic activity of curcumin or curcumin-Cu (II) compared to normal cells, and the IC_50_ of curcumin towards CCRF-CEM cells was 8.68 µM, and that of curcumin-Cu (II) was 8.14 µM. The cell cycle distribution of curcumin-treated CCRF-CEM cells was analyzed by flow cytometry. DNA damage induced by oxidants such as curcumin-Cu (II) ions is considered as one of the main causes of cell inactivation. Therefore, we analyzed the effect of curcumin on DNA damage by agarose gel electrophoresis and atomic force microscopy (AFM). Gel electrophoresis analyses showed that curcumin or Cu (II) alone failed to cause DNA damage in pBR322 plasmid DNA as compared with the normal plasmid. However, DNA plasmids were mostly damaged after treatment with curcumin of different concentrations in the presence of Cu (II). Two forms were observed by means of AFM: closed circular plasmids and linear plasmids. DNA damage induced by a combination of curcumin and Cu (II) was also found by agarose gel electrophoresis, which was applied as control method to verify the results obtained by AFM.

## 1. Introduction 

**Figure 1:**
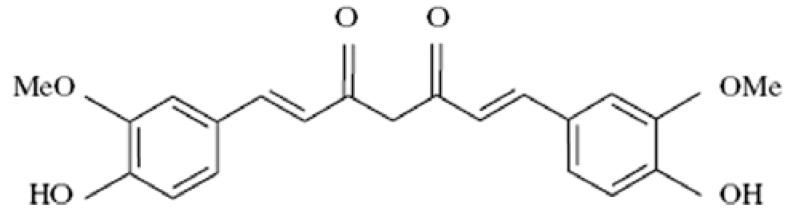
Structure of curcumin.

Curcumin is a natural pigment derived from turmeric (*Curcuma longa* L.), which has been known for its numerous pharmacological properties for many years [1,2,3]. It has the advantage of being nontoxic towards normal cells [4]. Traditionally, curcumin has been used as a coloring, food preservative and flavoring substance (Figure 1). 

Curcumin has been considered as one of the most promising chemopreventive agents against a variety of human cancers [5]. It may inhibit proliferation of cancer cells by cell cycle arrest and by inducing apoptosis [6,7]. Human leukemia is one of the commonly diagnosed neoplasms and the major leading cause of human death. The development of drug resistance and severe side effects of standard anticancer drugs necessitate the search for novel treatment options for this disease. The activity of drugs from traditional Chinese medicines including curcumin towards human CCRF-CEM leukemia cells has been reported [8]. 

**Figure 2:**
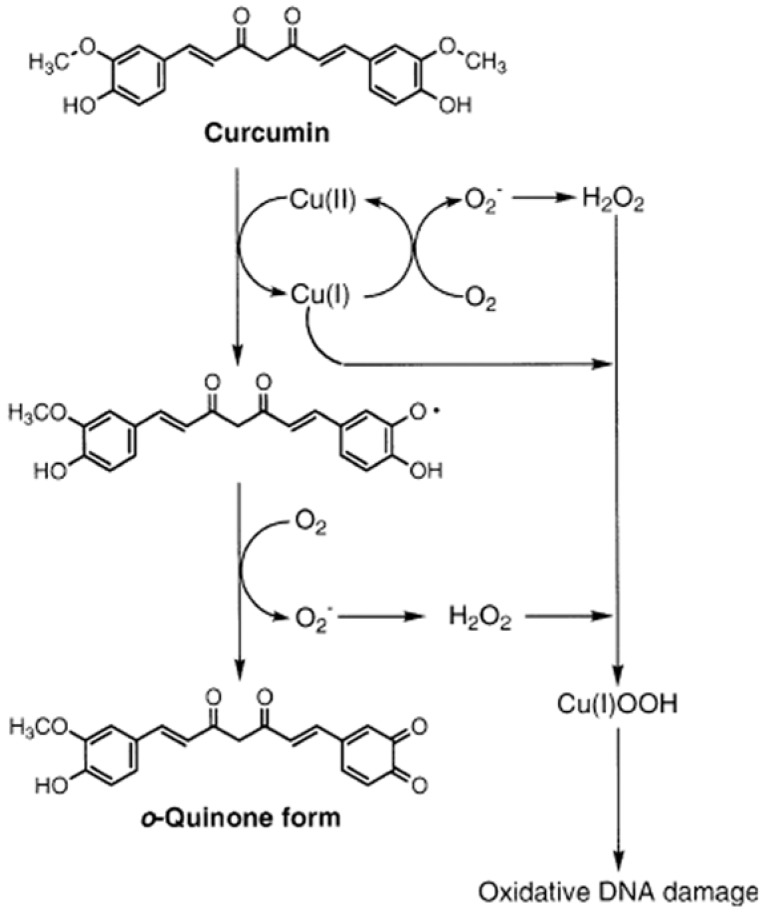
Proposed mechanism for curcumin–Cu (II) induced DNA damage.

DNA damage represents a crucial step in carcinogenesis, and the restoration of the DNA damage response is a promising strategy both to prevent cancer development and to improve response to conventional therapy [9]. The prooxidant action of plant-derived phenolics rather than their antioxidant action may play an important role in their anticancer and apoptosis-inducing properties [10]. Curcumin is a polyphenol [11], which can generate reactive oxygen species as a prooxidant agent in the presence of copper ions in cells, resulting in DNA damage and apoptotic cell death [12]. A possible mechanism of DNA damage induced by curcumin in the presence of Cu (II) was reported [13] and is shown in Figure 2.

In the present study, the cytotoxic activity of curcumin towards CCRF-CEM and Vero cells was determined by the MTT assay, and the cell cycle of CCRF-CEM cells treated with curcumin was analyzed by flow cytometry. Moreover, DNA damage induced by curcumin in the presence of Cu (II) was investigated with atomic force microscopy (AFM). Curcumin-induced DNA double-strand breaks have been determined by gel electrophoresis in previous study [12]. However, AFM has not been used to examine DNA damage induced by curcumin as of yet. Our study showed that DNA double-strand breaks were induced after exposure to curcumin by applying AFM as novel detection technique for DNA damage. Agarose gel electrophoretic analysis was used as control method.

## 2. Results and Discussion

### 2.1. Effect of Curcumin and Curcumin-Cu (II) on Cell Viability

**Figure 3:**
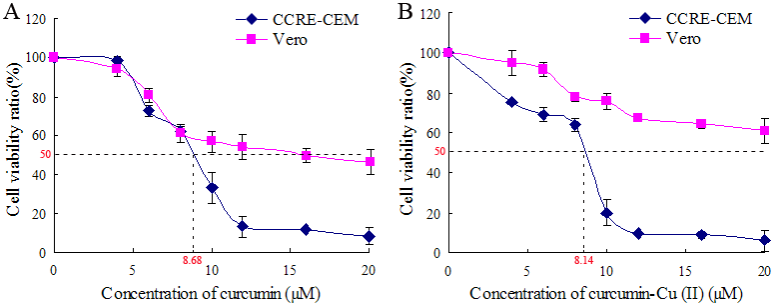
Effects of curcumin and curcumin-Cu (II) on the viability of CCRF-CEM and Vero cells as determined by the MTT assay. A: Treatment with curcumin; B: Treatment with curcumin-Cu (II). The values for each concentration tested represent the average (mean ± SD) from eight replicate wells and are representative of three separate experiments.

The effects of curcumin and curcumin-Cu (II) on the viability of CCRF-CEM and Vero cells were determined by the MTT assay. Figure 3A showed the effects of curcumin on cell viability after 48 h of drug exposure, the cell viability significantly decreased in ranges from 0 to 20 µM. Figure 3B showed that there was also a decrease in the viability in individual cell line with increasing concentration of curcumin-Cu (II) treatment. Curcumin-Cu (II) showed no obviously different sensitivity towards CCRF-CEM cells compared with curcumin treatment. The IC_50_ of curcumin-Cu (II) towards CCRF-CEM cells was 8.14 µM, and that of curcumin was 8.68 µM. The IC_50_ values of curcumin and curcumin-Cu (II) towards Vero cells were >15 µM. Those data indicated that curcumin and curcumin-Cu (II) had higher cytotoxic activity towards CCRF-CEM, but both of them had relatively lower cytotoxic activity with normal cells. It was testified tumor cells were more sensitive to curcumin and curcumin-Cu (II) compared with normal cells. The tumor cells’ differences in membrane structure, protein composition and bigger size against normal cells could be responsible for more sensitivity [14].

### 2.2. Cell Cycle Analysis by Flow Cytometry

**Figure 4:**
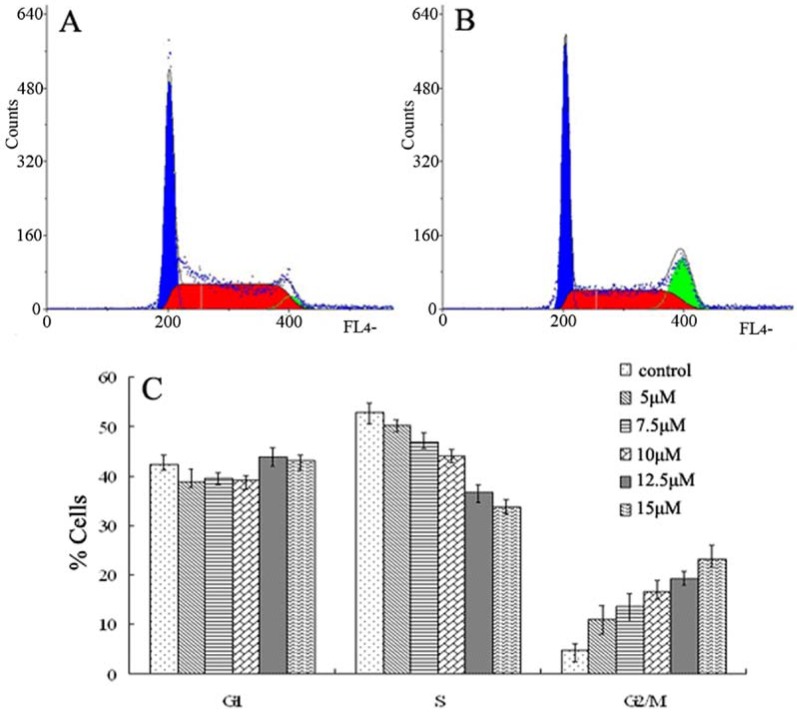
Cell cycles distribution of CCRF-CEM cells after treatment with different concentrations of curcumin for 48 h. Flow cytometric analysis of CCRF-CEM cell cycle distribution, (A) Untreated control cells; (B) Cells treated with 15 μM curcumin; (C) The results were analyzed by Mod Fit LT 3.0. Data are presented as mean ± SD (n = 3). **p < 0.01, *p < 0.05; p value compared with the control group (0 μM).

To evaluate the cell cycle phase distribution of CCRF-CEM cells with or without curcumin treatment, the DNA content was measured by flow cytometry. Exposure to curcumin from 5 to 15 µM for 48 h caused an increase of the G_2_/M phase population from 11.0% to 23.1%, as compared to control cells that showed a fraction of 4.87% G_2_/M phase cells (Figure 4A). This was accompanied by a significant decrease in the percentage of S phase cells, whereas the fraction of G1 phase cells was mainly unchanged (Figure 4C). This result demonstrates that curcumin induces growth-inhibitory effects at least in part via G2/M phase arrest.

### 2.3. Plasmid DNA Analysis by Agarose Gel Electrophoresis

**Figure 5:**
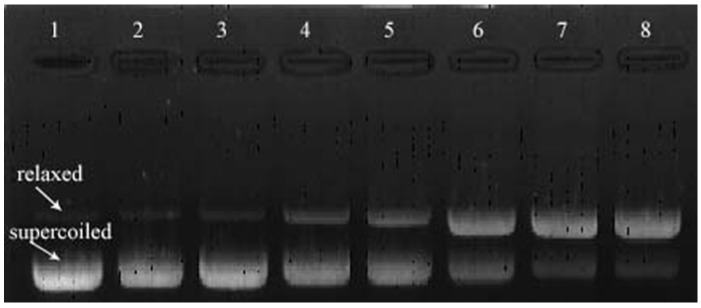
Agarose gel electrophoretic patterns of plasmid DNA treated with curcumin in the presence of Cu (II) ions (0.2 mM). pBR322 plasmid DNA (0.5 μg) was incubated for 1 h at 37 ºC in the presence of the following additives: lane 1, no addition (DNA control); lane 2, 0.2 mM curcumin without copper; lane 3, copper without curcumin; lanes 4–8, copper plus curcumin at different concentrations, 0.01, 0.025, 0.05, 0.1, and 0.2 mM, respectively.

The destruction of supercoiled pBR322 plasmid DNA and the formation of open circular DNA were used to assess DNA strand breakage. Figure 5 showed the DNA damage induced by curcumin and/or Cu (II). pBR322 DNA plasmids treated with curcumin (0.2 mM) or Cu (II) (0.2 mM) alone were not damaged. However, DNA was severely damaged by curcumin in the presence of Cu (II). The fraction of relaxed DNA plasmids increased in a concentration-dependent manner as compared to the control. It is suggested that curcumin induces DNA damage in the presence of Cu (II) and breaks supercoiled DNA in a concentration-dependent manner.

### 2.4. AFM Imaging of Plasmid DNA 

**Figure 6:**
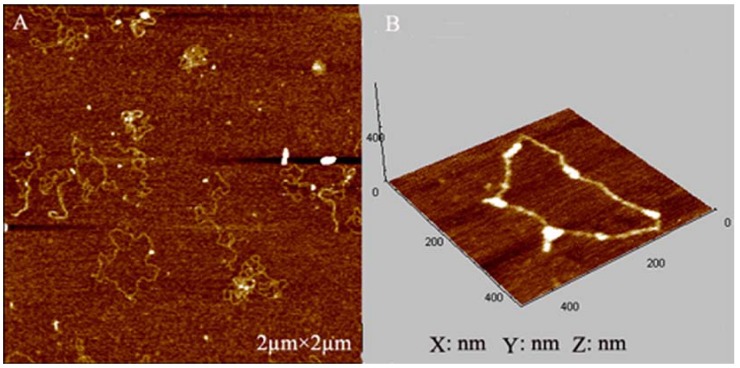
Untreated pBR332 DNA plasmid. A: Two-dimensional representation. Size = 2 µm × 2 µm; B: Three-dimensional representation. Size = 400 nm × 400 nm × 400 nm.

**Figure 7:**
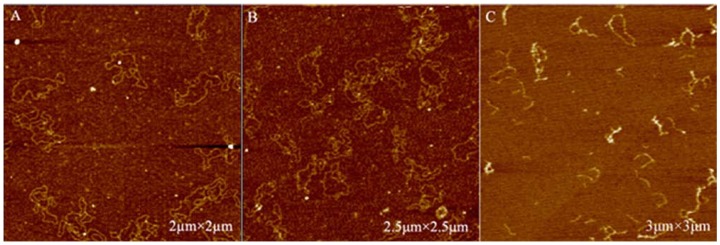
AFM imaging of pBR332 DNA plasmids treated with curcumin and/or Cu (II). A: Plasmid 0.05 µg plus 0.02 mM Cu (II) for 2 h; B: Plasmid 0.05 µg plus 0.02 mM curcumin for 2 h; C: Plasmid 0.05 µg plus 0.02 mM Cu (II) plus 0.02 mM curcumin for 2 h.

In this study, AFM imaging was applied for DNA damage analysis before and after being treated with curcumin and/or Cu (II). Figure 6 showed the AFM images of untreated DNA. Almost all the untreated control DNA molecules appeared in a closed circular form. Exposure to 0.02 mM Cu (II) or 0.02 mM curcumin alone did not affect the native form of plasmid DNA. As can be seen in Figure 7A, B, DNA was still supercoiled. However, two forms of plasmid DNA such as closed circular and linear plasmids were observed in the test samples induced by curcumin in the presence of Cu (II) (Figure 7C).

Acute lymphoblastic leukemia, a malignant disorder of lymphoid progenitor cells, affects both children and adults, with peak prevalence between the age of two and five years [15]. It is thought to originate from various important genetic lesions in blood-progenitor cells that are committed to differentiate in the T-cell or B-cell pathway [16]. Through synthetic compounds were applied to prevent cancer, naturally occurring dietary substances are more preferred by patients for cancers chemoprevention. Curcumin is a low molecular weight polyphenol, derived from the herbal remedy and dietary spice turmeric. The chemopreventive potential of curcumin is well documented in the literature [17]. Our data showed that curcumin exhibited notable antiproliferative activity towards CCRF-CEM cells. Blockade of the cell cycle is regarded as an effective strategy for the development of novel treatment options for cancer [18,19]. Curcumin inhibits proliferation of cancer cells by arresting them in various phases of the cell cycle [20,21]. In our study, curcumin effectively arrests CCRF-CEM cells in the G_2_/M phase. Therefore, curcumin may be selected to prevent acute lymphoblastic leukaemia in clinic.

Curcumin possesses high antioxidant activity [22]. However, an antioxidant can become a prooxidant, accelerate lipid peroxidation, and induce DNA damage under special conditions [23]. Contrary to the antioxidant nature of curcuminoids, curcumin can also act as a prooxidant by forming reactive oxygen species through the reduction of copper causing activation of oxygen molecules. Curcumin/copper-dependent formation of hydroxyl radicals can play a principal role in DNA damage [12]. DNA damage is related to both apoptosis and carcinogenesis. If DNA damage is weak, the cellular response allows the repair of damage. However, if the damage failed to be repaired, mutagenic lesions could be propagated and might lead to carcinogenesis [24]. Curcumin is not only an antioxidant, but also a pro-oxidant in the presence of Cu (II) and can induce DNA damage in cancer cells, finally leading to the apoptosis of cancer cells. In the present study, gel electrophoresis and AFM were used to investigate DNA damage induced by curcumin in the presence of Cu (II). We found that AFM is a suitable technique to provide detailed information on DNA strand break formation in the nanometer range under physiological conditions. This is in accord with data of Su *et al*. [25]. We demonstrated that curcumin in the presence of Cu (II) causes severe DNA damage. This indicates that DNA damage may also represent a mechanism of action of curcumin towards CCRF-CEM cells. This merits will be further investigated in the future. 

## 3. Experimental Section

### 3.1. Growth of Cell, Plasmid DNA and Chemicals

The human T-cell leukemia CCRF-CEM cell line was kindly provided by Prof. Dr. Thomas Efferth (German Cancer Research Center, Heidelberg, Germany) and was maintained in RPMI 1640 medium supplemented with 10% fetal bovine serum, 100 U/mL penicillin and 100 μg/mL streptomycin. African green monkey kidney epithelial cell (Vero) was purchased from Harbin Medical University, China and was maintained in DMEM medium. The cells were kept at 37 °C in an atmosphere containing 5% CO_2_.

Curcumin (purity ≥ 98%) was purchased from Sigma-Aldrich (St. Louis, MO, USA). A 100 mM stock solution of curcumin was prepared in dimethyl sulfoxide (DMSO) and stored at −80 °C, and the final concentration of DMSO in the test medium did not exceed 0.1% v/v. MTT (3-(4,5-dimethylthiazol-2yl)2,5-diphenyltetrazolium bromide) was from Sigma-Aldrich. Sulfate heptahydrate Cu (II) was freshly prepared for each experiment. pBR322 DNA was purchased from TOYOBO Co., Ltd. (Osaka, Japan). Deionized water was used in all experiments.

### 3.2. MTT Assay

The effects of curcumin and curcumin-Cu (II) on the viability of CCRF-CEM and Vero cells were determined by the MTT assay (26). In each experiment, exponentially growing cells were plated in 100 μL aliquots of growth medium into 96-well plates at 10^5^ cells per well, respectively, and incubated for 24 h. For loading of curcumin, the cells (in 96-well plates) were incubated with curcumin (at a concentration of 0, 4, 6, 8, 10, 12, 16 and 20 μΜ; eight wells per concentration), another group of cells (in 96-well plates) were incubated with curcumin-Cu (II) (at a concentration of 0, 4, 6, 8, 10, 12, 16 and 20 μΜ; eight wells per concentration). After 48 h incubation, MTT solution (5 mg/mL) was then added to each well, and the formazan precipitate was dissolved in 100 μL dimethyl sulfoxide after 4 h incubation, and then the absorbance was measured in an ELISA reader (Thermo Molecular Devices Co., Union City, USA) at 570 nm. The cell viability ratio was calculated by the following formula: Cell viability ratio (%) = $\frac{{\mathrm{OD}}_{\mathrm{treated}}}{{\mathrm{OD}}_{\mathrm{control}}}$ × 100%. The IC_50_ (the concentration reducing cell viability by 50%) was calculated from the concentration-response and expressed as μM.

### 3.3. Cell Cycle Analysis

CCRF-CEM cells were seeded in six well plates in standard growth medium at a density of 10^5^ cells per well. After incubation overnight for 24 h, the cells were treated with curcumin at different concentrations (5, 7.5, 10, 12.5 and 15 μM) and incubated for 48 h. Then, cells were centrifuged and washed with PBS and stained with 50 μg/mL DAPI (Partec, Münster, Germany) and analyzed by means of a FACS flow cytometer (Partec). The percentages of cells in G_0_/G_1_ phase, S phase and G_2_/M phase were analyzed with FACScan and CellQuest software (Becton Dickinson, Mountain View, CA, USA).

### 3.4. DNA Damage Induced by Curcumin

#### 3.4.1. Plasmid DNA Analysis by Agarose Gel Electrophoresis

The induction of DNA strand breakage by curcumin was assessed by measuring the conversion of supercoiled pBR322 plasmid DNA to open circular and linear forms or to further fragmentation by gel electrophoresis [27]. pBR322 DNA was treated with curcumin and/or Cu (II) in 10 mM Tris-HCl buffer at pH 7.4 and 37 ºC for 1 h. The mixture of 10 μL contained 0.5 μg of pBR322, 0.2 mM Cu (II) and curcumin of a series of dilution (0.2, 0.1, 0.05, 0.025 and 0.01 mM). After incubation, the samples were loaded on 0.8% agarose gel and electrophoresed for 30 min at 100 V at room temperature. After electrophoresis, the gel was stained with ethidium bromide and visualized on a UV-transilluminator (Image Master VDS-CL, Tokyo, Japan). 

#### 3.4.2. Atomic Force Microscope (AFM) Imaging of Plasmid DNA

AFM was performed using a commercial AFM system (PicoPlus, Molecular Imaging, Tempe, AZ, USA) operated in the tapping mode at room temperature. Resonance frequencies of 60–90 kHz were used. The scan rates were 2–4 Hz. To prepare the test samples for AFM, 0.05 µg pBR322 was treated with 0.02 mM curcumin and/or 0.02 mM Cu (II) at 37 ºC for 2 h. Then, the DNA solution (10 µL) was deposited on a 1-(3-aminopropyl)silatrane-functionalyzed mica (APS-mica) surface, and allowed to adsorb for 5 min. APS-mica was prepared as described by Shlyakhtenko *et al*. [28]. The sample was rinsed and air dried before imaging. This procedure resulted in a stable attachment of DNA to the mica surface that could be imaged for hours by AFM.

## 4. Conclusions

In summary, our study demonstrates that CCRF-CEM human T-cell leukemia cells were more sensitive to the cytotoxic activity of curcumin or curcumin-Cu (II) compared with normal cells. Curcumin induced growth-inhibitory effects via G2/M phase arrest. Gel electrophoresis and AFM analyses showed that curcumin or Cu (II) alone did not lead to DNA lesion, whereas a combination of both agents led to a rupture of plasmid DNA. These results testified that the cytotoxic activity of curcumin was attributed to its pro-oxidant activity. The present study contributed to the scientific basis for the application of curcumin in food and medicinal industry.
